# Insulin resistance in school-aged girls with overweight and obesity is strongly associated with elevated white blood cell count and absolute neutrophil count

**DOI:** 10.3389/fendo.2022.1041761

**Published:** 2022-11-07

**Authors:** Hong-Mei He, Lu Zhang, Na Qiu, Ze-Tao Zhou, Ka Zhang, Yan Li, Hao-Bo Chen, Jia-Ning Xu

**Affiliations:** ^1^ Department of Preventive Medicine, School of Basic Medical Sciences, Henan University, Kaifeng, China; ^2^ Institute of Biomedical Informatics, Bioinformatics Center, Henan University, Kaifeng, China; ^3^ Henan Provincial Engineering Center for Tumor Molecular Medicine, Kaifeng, China

**Keywords:** pediatric obesity, sex, insulin resistance, white blood cell count, absolute neutrophil count

## Abstract

**Background:**

The primary objective of the study was to discuss the sex differences in insulin resistance-induced changes in metabolic and inflammatory markers in school-aged children with overweight and obesity.

**Methods:**

A cross-sectional study of 800 children aged seven and twelve years was performed. Questionnaires, anthropometric data and fasting blood samples were collected.

**Results:**

Children with overweight and obesity showed statistically significant differences in multiple metabolic and inflammatory markers compared with children with normal BMI. The correlation coefficient (*r*) between white blood cell count, absolute neutrophil count, fasting plasma insulin, HOMA-IR, HOMA-β, triglyceride, HDL-C, triglyceride/HDL ratio, alanine transaminase, serum uric acid, systolic blood pressure and BMI were higher in all children, but the linear relationships between white blood cell count, absolute neutrophil count and BMI were stronger in girls with overweight and obesity than in boys with overweight and obesity. Subsequently, HOMA-IR was shown to be more strongly associated with increased white blood cell count and absolute neutrophil count in school-aged girls with overweight and obesity by partial correlation analysis and the multiple linear regression analysis.

**Conclusions:**

Elevated white blood cell count and absolute neutrophil count in children with overweight and obesity, especially girls, can serve as markers of insulin resistance.

## Introduction

Obesity of children and adolescents is a worldwide health problem that is becoming more prevalent in low- and middle-income countries, as in many high-income countries. According to the assessment of the World Obesity Federation, 206 million children and adolescents aged 5-19 will be obese by 2025, and by 2030 there will be 254 million. In 42 countries with an estimated 1 million children with obesity in 2030, China ranks first, followed by India, the United States, Indonesia and Brazil, and only seven of the top 42 are high-income countries (1). About a fifth of all children are overweight or obese according to the Chinese sex-age-specific BMI cutoff points in China. By 2030, the prevalence of overweight and obesity in school-age children may come to 31.8%, or about 58.92 million (2). Obesity is often associated with insulin resistance and is the leading cause of insulin resistance in children. The main cause of children’s insulin resistance is the typical lipid distribution pattern, that is, increased deposition of lipids in insulin sensitive tissue such as the liver, skeletal muscle and viscera. This lipid deposition pattern is also associated with the infiltration of immune system cells into intra-abdominal tissues, inducing systemic low-grade inflammation (3).

Reavan GM argues that insulin resistance is crucial in the pathogenesis of type 2 diabetes, hypertension, and coronary heart disease (4). Insulin resistance manifests as hyperinsulinemia and is a driver of dyslipidemia, high blood pressure and altered glucose metabolism (5). Therefore, insulin resistance can cause changes in multiple metabolic and systemic inflammatory markers (6–8). However, this effect may differ between boys and girls, and there are few such studies. Therefore, the aim of the present study was to determine if sex differences existed in insulin resistance-induced changes in metabolic and inflammatory markers in school-aged children who were overweight and obese.

## Materials and methods

### Study design and samples

This cross-sectional study was carried out in Kaifeng, Henan Province from October to November 2019 and July to September 2020, during which time it was interrupted due to the COVID-19 outbreak. Five primary schools were selected by cluster sampling from 33 primary schools of a district in Kaifeng. According to the data from last year’s school physical examination, 1,297 children aged 7-12 years were investigated by simple random sampling. Eight hundred children completed questionnaires, anthropometric measures and laboratory tests, but the remainder did not take fasting blood samples because their parents or guardians did not agree or had no time to attend.

Inclusion criteria for the study samples were:

-All participants participated in the study voluntarily.
-They were students in grade 2 to 6 of primary school.
-No metabolic or endocrine disease.
Exclusion criteria for the study samples were:
-Any pathological changes, such as endocrine, metabolic or inadequate renal function, which may contribute to changes in dietary habits and nutrient intake.
-Infectious diseases and treatment with antibiotics.

Written informed consents were acquired from parents or guardians. This study was approved by the Ethics Committee of Henan University.

### Questionnaire survey

The demographic characteristics, lifestyle, diet, home environment, maternal pregnancy, feeding patterns in infancy and other risk factors were assessed with standardized questionnaires for students and parents. Under the guidance of well-trained investigators, the student questionnaire was completed in the school. The parents’ questionnaire was taken home and filled in by the parents. After all questionnaires are collected, if there are any problems, the investigators would call the parents to verify.

### Anthropometric studies

The height, weight and waist circumference of children wearing light clothing without shoes were measured with standard methods, and the data were accurate to 0.1 cm and 0.1 kg. The electronic sphygmomanometer (Omron HEM-7136) was used to measure the systolic and diastolic blood pressure for three times with an interval of 30 seconds. The average value was calculated for analysis. Body mass index (BMI) was calculated by dividing a child’s weight (in kilograms) by height (in square meters). The diagnosis of overweight and obesity is based on Chinese sex- and age-specific BMI criteria, that is, a BMI at or above the 85^th^ and 95^th^ percentile, respectively (9). The age-specific BMI Z-scores were calculated using WHO AnthroPlus software. According to BMI Z-scores, children were classified as: normal weight with Z-scores from −2 to +0.99, overweight from 1 to 1.99, obese from 2 to 2.99, and very obese ≥ 3 (10, 11).

### Hematological and clinical biochemical studies

Overnight fasting blood samples were collected for measuring blood routine examination, blood lipid level, liver function, kidney function, fasting blood glucose, fasting insulin and C-reactive protein. Insulin resistance and β-cell function were calculated from fasting blood glucose and fasting insulin by the homeostasis model assessment of insulin resistance (HOMA-IR index) (12). The neutrophil-to-lymphocyte ratio was determined by dividing the absolute neutrophil count by the absolute lymphocyte count, the platelet-to-lymphocyte ratio determined by dividing the platelet count by the absolute lymphocyte count, and the triglyceride/HDL ratio determined by dividing the triglyceride level by the high-density lipoprotein cholesterol (HDL-C) level (13–15).

### Statistical analysis

None of the quantitative variables were normally distributed. Quantitative variables and categorical variables are summarized as median (interquartile range) and number (percentage), respectively. Differences between participants with and without overweight/obesity or male and female were evaluated by the nonparametric Wilcoxon test for quantitative data and the chi-square test for categorical data. Although the quantitative variables in this study do not conform to a normal distribution, they can be regarded as approximately a normal distribution due to the large sample size. So the partial correlation analysis was used to analyze the linear relationship between BMI and the clinical indicators and between HOMA-IR and the clinical indicators adjusted for confounding factors. Multiple linear regression analysis showed a sex difference in the association of HOMA-IR and the white blood cell count/absolute neutrophil count in children who were overweight and obese.

All analyses were performed with SPSS 26.0 (IBM, Armonk, NY, USA), and a two-tailed *P*<0.05 was the level of statistically significant.

## Results

### Clinical characteristics of population

800 school-age children (474 boys and 326 girls) aged between 7 to 12 years were included in this study. According to the BMI Z-score established by the WHO, there were 161 children with normal weight and 639 children with overweight and obesity. According to Chinese sex- and age-specific BMI criteria, 181 children are of normal BMI and 619 are overweight or obese. There was no statistical difference between the two classification methods (*χ* ^2^ = 1.49, *P*=0.223). The classification method used in this study is Chinese criteria.

The clinical characteristics of the normal BMI and overweight/obesity participants are presented in **Table 1**. In addition to absolute lymphocyte counts, absolute basophils count, fasting plasma glucose, aspartate transaminase, blood urea nitrogen and serum creatinine, other clinical indicators were statistically different between children with normal BMI and those who were overweight and obese.

**Table 1:** Clinical characteristics of school-age children with normal BMI and overweight/obesity.

Variables	Normal BMI (n=181)	Overweight/obesity (n=619)	*P*
Sex			
Male	96 (20.25)	378 (79.75)	0.053
Female	85 (26.07)	241 (73.93)	
Age (years)	9.40 (8.70-10.65)	10.20 (9.20-10.90)	<0.001
BMI Z-score	1.63(0.67-2.56)	2.08 (1.40-2.54)	<0.001
White blood cell count (10^9^/L)	6.03 (5.23-6.91)	7.15 (6.08-8.44)	<0.001
Absolute neutrophil count (10^9^/L)	2.92 (2.32-3.58)	3.79 (3.05-4.73)	<0.001
Absolute lymphocyte counts (10^9^/L)	2.53 (2.19-2.95)	2.64 (2.18-3.18)	0.065
Absolute monocyte count (10^9^/L)	0.34 (0.28-0.41)	0.38 (0.32-0.47)	<0.001
Absolute eosinophil count (10^9^/L)	0.11 (0.07-0.19)	0.13 (0.09-0.21)	0.028
Absolute basophils count (10^9^/L)	0.03 (0.02-0.03)	0.03 (0.02-0.04)	0.781
Red blood cell count (10^12^/L)	4.74 (4.55-4.92)	4.84 (4.65-5.05)	<0.001
Hemoglobin concentration (g/L)	135.00 (129.25-140.00)	136.00 (131.00-141.00)	0.042
Hematocrit (%)	40.75 (39.13-42.08)	41.30 (39.90-42.70)	<0.001
Mean corpuscular volume (fL)	85.70 (83.90-87.88)	85.00 (82.80-87.30)	0.005
Mean corpuscular hemoglobin (pg)	28.40 (27.73-29.10)	28.10 (27.30-28.80)	<0.001
Mean corpuscular hemoglobin concentration (g/L)	332.00 (328.00-335.00)	330.00 (325.00-334.00)	<0.001
Platelet count (10^9^/L)	287.00 (248.25-323.00)	312.00 (274.00-354.25)	<0.001
Neutrophil-to-lymphocyte ratio	1.16 (0.91-1.44)	1.42 (1.11-1.81)	<0.001
Platelet-to-lymphocyte ratio	114.79 (93.39-136.48)	119.25 (100.32-141.90)	0.004
Fasting plasma insulin (mIU/L)	5.81 (4.14-8.15)	12.38 (8.23-19.16)	<0.001
Fasting plasma glucose (mmol/L)	4.93 (4.73-5.21)	4.98 (4.75-5.29)	0.227
HOMA-IR	1.28 (0.90-1.88)	2.74 (1.80-4.25)	<0.001
HOMA-β	78.59 (59.15-112.49)	164.53 (111.57-270.58)	<0.001
Total cholesterol (mmol/L)	3.84 (3.39-4.29)	4.08 (3.63-4.56)	<0.001
Triglyceride (mmol/L)	0.81 (0.62-1.08)	1.13 (0.84-1.53)	<0.001
High-density lipoprotein cholesterol (mmol/L)	1.38 (1.24-1.57)	1.22 (1.09-1.37)	<0.001
Low-density lipoprotein cholesterol (mmol/L)	2.23 (1.92-2.51)	2.41 (2.13-2.78)	<0.001
Triglyceride/HDL ratio	0.57 (0.43-0.75)	0.94 (0.64-1.35)	<0.001
Total bilirubin (μmol/L)	10.99 (9.08-13.52)	10.01 (8.20-12.53)	0.001
Aspartate transaminase (U/L)	22.85 (20.20-26.10)	23.00 (19.30-28.10)	0.697
Alanine transaminase (U/L)	12.25 (10.33-15.60)	18.30 (13.60-31.30)	<0.001
Blood urea nitrogen (mmol/L)	4.07 (3.48-4.97)	4.03 (3.33-4.70)	0.314
Serum creatinine (μmol/L)	43.90 (40.53-48.50)	44.05 (40.50-48.10)	0.928
Serum uric acid (μmol/L)	298.17 (255.15-353.71)	366.24 (316.76-423.21)	<0.001
C-reactive protein (mg/L)	4.08 (3.80-4.37)	4.39 (2.21-4.47)	0.001
Systolic blood pressure (mmHg)	101.33 (95.67-107.33)	109.00 (102.67-116.25)	<0.001
Diastolic blood pressure (mmHg)	70.33 (66.33-74.33)	72.33 (67.42-77.67)	0.001

BMI, body mass index; HOMA-β, homeostasis model assessment of beta-cell function; HOMA-IR, homeostasis model assessment of insulin resistance.

### Linear relationship between BMI and clinical indicators

Partial correlation analysis was used to show a linear relationship between BMI and clinical indicators adjusted for age and sex (**Table 2**). Because the correlation coefficients *r* between white blood cell count, absolute neutrophil count, fasting plasma insulin, HOMA-IR, HOMA-β, triglyceride, HDL-C, triglyceride/HDL ratio, alanine transaminase, serum uric acid, systolic blood pressure and BMI were stronger (*r*>0.3, *P*<0.05), these linear relationships were further explored for sex differences.

**Table 2:** Partial correlation analysis of BMI and clinical indicators adjusted for age and sex in all children.

Variables	BMI	
	*r*	*P*
White blood cell count (10^9^/L)	0.32	<0.001
Absolute neutrophil count (10^9^/L)	0.31	<0.001
Absolute monocyte count (10^9^/L)	0.26	<0.001
Absolute eosinophil count (10^9^/L)	0.003	0.926
Red blood cell count (10^12^/L)	0.20	<0.001
Hemoglobin concentration (g/L)	0.06	0.107
Hematocrit (%)	0.14	<0.001
Mean corpuscular volume (fL)	-0.20	<0.001
Mean corpuscular hemoglobin (pg)	-0.18	<0.001
Mean corpuscular hemoglobin concentration (g/L)	-0.19	<0.001
Platelet count (10^9^/L)	0.26	<0.001
Neutrophil-to-lymphocyte ratio	0.16	<0.001
Platelet-to-lymphocyte ratio	0.06	0.097
Fasting plasma insulin (mIU/L)	0.54	<0.001
HOMA-IR	0.50	<0.001
HOMA-β	0.56	<0.001
Total cholesterol (mmol/L)	0.14	<0.001
Triglyceride (mmol/L)	0.37	<0.001
High-density lipoprotein cholesterol (mmol/L)	-0.37	<0.001
Low-density lipoprotein cholesterol (mmol/L)	0.21	<0.001
Triglyceride/HDL ratio	0.41	<0.001
Total bilirubin (μmol/L)	-0.14	<0.001
Alanine transaminase (U/L)	0.34	<0.001
Serum uric acid (μmol/L)	0.44	<0.001
C-reactive protein (mg/L)	0.10	0.006
Systolic blood pressure (mmHg)	0.40	<0.001
Diastolic blood pressure (mmHg)	0.22	<0.001

BMI, body mass index; HOMA-β, homeostasis model assessment of beta-cell function; HOMA-IR, homeostasis model assessment of insulin resistance.

### Sex differences in the linear relationships between BMI, HOMA-IR and clinical indicators

Sex differences in the linear relationships between white blood cell count, absolute neutrophil count, fasting plasma insulin, HOMA-IR, HOMA-β, triglyceride, HDL-C, triglyceride/HDL ratio, alanine transaminase, serum uric acid, systolic blood pressure and BMI adjusted for age were analyzed in school-age children with normal BMI and who were overweight and obese. We found the linear relationships between white blood cell count, absolute neutrophil count and BMI that were stronger in girls than in boys who were overweight and obese (**Table 3**), but these sex differences were not seen in normal BMI children (**Supplementary Table 1**).

**Table 3:** Sex differences in partial correlation analysis of BMI and clinical indicators adjusted for age in children with overweight and obesity.

Variables	Boy’s BMI (n=378)		Girl’s BMI (n=241)	
	*r*	*P*	*r*	*P*
White blood cell count (10^9^/L)	0.17	0.001	0.37	<0.001
Absolute neutrophil count (10^9^/L)	0.14	0.007	0.35	<0.001
Fasting plasma insulin (mIU/L)	0.47	<0.001	0.48	<0.001
HOMA-IR	0.43	<0.001	0.44	<0.001
HOMA-β	0.49	<0.001	0.52	<0.001
Triglyceride (mmol/L)	0.28	<0.001	0.29	<0.001
High-density lipoprotein cholesterol (mmol/L)	-0.32	<0.001	-0.28	<0.001
Triglyceride/HDL ratio	0.33	<0.001	0.32	<0.001
Alanine transaminase (U/L)	0.31	<0.001	0.35	<0.001
Serum uric acid (μmol/L)	0.35	<0.001	0.46	<0.001
Systolic blood pressure (mmHg)	0.33	<0.001	0.38	<0.001

BMI, body mass index; HOMA-β, homeostasis model assessment of beta-cell function; HOMA-IR, homeostasis model assessment of insulin resistance.

In this study, HOMA-IR was found to be higher in girls who were overweight and obese than in boys who were overweight and obese (*P*<0.001), with a HOMA-IR in girls of 3.06 (2.12-4.76) and in boys of 2.56 (1.68-3.93). A more pronounced linear relationship was found between white blood cell count, absolute neutrophil count and HOMA-IR in girls who were overweight and obese (**Table 4**).

**Table 4:** Sex differences in partial correlation analysis of HOMA-IR and clinical indicators adjusted for age in children with overweight and obesity.

Variables	Boy’s HOMA-IR (n=378)		Girl’s HOMA-IR (n=241)	
	*r*	*P*	*r*	*P*
White blood cell count (10^9^/L)	0.12	0.025	0.27	<0.001
Absolute neutrophil count (10^9^/L)	0.09	0.071	0.29	<0.001
Triglyceride (mmol/L)	0.31	<0.001	0.25	<0.001
High-density lipoprotein cholesterol (mmol/L)	-0.10	0.043	-0.13	0.039
Triglyceride/HDL ratio	0.27	<0.001	0.22	0.001
Alanine transaminase (U/L)	0.23	<0.001	0.23	<0.001
Serum uric acid (μmol/L)	0.17	0.001	0.14	0.028
Systolic blood pressure (mmHg)	0.29	<0.001	0.32	<0.001

HOMA-IR, homeostasis model assessment of insulin resistance.

### Sex differences in the association between HOMA-IR and white blood cell count/absolute neutrophil count

Multiple linear regression analysis showed that HOMA-IR was associated with white blood cell count (*β*=0.18, *P*<0.001) and absolute neutrophil count (*β*=0.15, *P*<0.001) when adjusted for age in girls who were overweight and obese, but the association was less pronounced in boys who were overweight and obese (**Table 5**).

**Table 5:** Sex differences in multiple linear regression analysis of HOMA-IR and white blood cell count/absolute neutrophil count adjusted for age in children with overweight and obesity.

Independent variable	White blood cell count (10^9^/L)	Absolute neutrophil count (10^9^/L)
	*β* (95%CI), *P*	*β* (95%CI), *P*
Male (n=378)		
HOMA-IR	0.09 (0.01-0.16), 0.024	0.05 (-0.004-0.11), 0.069
Age	-0.04 (-0.22-0.15), 0.711	0.01 (-0.14-0.16), 0.892
Female (n=241)		
HOMA-IR	0.18 (0.10-0.27), <0.001	0.15 (0.09-0.21), <0.001
Age	-0.10 (-0.30–0.11), 0.349	0.04 (-0.11-0.19), 0.643

HOMA-IR, homeostasis model assessment of insulin resistance.

## Discussion

We determined if sex differences existed in insulin resistance-induced changes in metabolic and inflammatory markers in children who were overweight and obese through data from physical measurements and haematological tests of 800 school-age children in this study. We found that HOMA-IR was more strongly associated with increased white blood cell count and absolute neutrophil count in school-aged girls who were overweight and obese.

Insulin resistance is defined in physiological terms as requiring higher concentrations of insulin to trigger the physiological effects formerly induced by lower concentrations. Obesity is the leading cause of insulin resistance in children, and insulin resistance is closely associated with multiple cardiovascular risk factors and metabolic disorders, such as dyslipidemia, impaired glucose tolerance, type 2 diabetes, hyperuricemia, and elevated transaminases. Currently, there is no universally accepted definition of insulin resistance because there is no standardized analytical method for measuring plasma insulin. The “gold standard” method for measuring systemic insulin sensitivity is the euglycemic-hyperinsulinemic clamp (16). However, due to the complexity of the procedure, this methodology is used only in scientific research but not in clinical application. So HOMA-IR has been established and widely used as a substitute indicator of whole body insulin resistance (17).

In addition to routine blood tests and metabolism-related indicators, this study also assessed the association of novel markers with insulin resistance, such as C-reactive protein, neutrophil-to-lymphocyte ratio, platelet-to-lymphocyte ratio and triglyceride/HDL ratio. Fasting insulin levels are higher in children who were overweight and obese when compared to subjects with normal BMI, and the levels of HOMA-IR and HOMA-B were also higher. By partial correlation analysis, white blood cell count and absolute neutrophil count were more strongly correlated with BMI and HOMA-IR in girls who were overweight and obese. Subsequently, multiple linear regression analysis also demonstrated that HOMA-IR significantly increased white blood cell counts and absolute neutrophil count in girls who were overweight and obese.

Men and women have different energy needs, and there are sex differences in human metabolism (18). The biological differences between men and women lead to different physiological responses to exercise, including height, weight, fat mass, lean muscle mass and hormone levels. During prolonged exercise, women showed a greater ability to oxidize lipids as a fuel source, while men oxidized more protein and carbohydrates (19). Adipose tissue, considered a major storage site for excess energy, is now recognized as an endocrine organcapable of producing and releasing bioactive compounds involved in chronic inflammatory and pathological metabolic processes associated with obesity (20). Recent evidence have shown that excess adipose tissue is tightly associated with increased adipokine release, immune cell infiltration, and the progress of low-grade systemic inflammation from childhood to adulthood (21). Multiple studies have shown that the major cellular component of adipose tissue is adipocytes, which are sustained by an extracellular matrix interspersed with preadipocytes, fibroblasts, endothelial cells and immune cells (22, 23). Especially, the strong local presence of leukocytes such as macrophages, mast cells, natural killer cells, neutrophils, monocytes, and T and B lymphocytes led human adipose tissue defined as an immune organ that maintained delicate immune homeostasis (24, 25).

A complete blood count is an inexpensive and readily available blood test. Obesity is associated with hematologic abnormalities (26). Herishanu et al. analyzed 327 patients with persistent leukocytosis in a hematological clinic and found that 15% of the patients were asymptomatic and obese, most of whom were middle-aged females with mild leukocytosis, it is characterized by increased neutrophilia with elevated acute-phase reactants (C-reactive protein and erythrocyte sedimentation rate) (27). Raghavan et al. similarly noted that BMI was associated with white blood cell count and neutrophil count within the physiological range in obese women (28). Obesity-related leukocytosis is significantly predominant in women, and the etiology of this leukocytosis may be multifactorial. Sex-specific pathways of inflammation that affect obesity and metabolic syndrome have been identified. In patients with metabolic syndrome, women have lower concentrations of anti-inflammatory adiponectin. However, in men, metabolic syndrome is associated with increased monocyte-derived circulating cytokines (mainly IL-6) and hyperresponsive circulating immune cells (29). In addition, in females, inflammation may be limited by estrogen (30). Importantly, we found that in school-aged girls who were overweight and obese, increased white blood cell count and absolute neutrophil count were strongly associated with increased HOMA-IR.

There are some limitations in the current study, including the cross-sectional study design, the lack of assessment of a wider range of inflammatory biomarkers, such as IL-6 and TNF-α, and the physical changes of secondary sexual characteristics during puberty were not evaluated.

## Conclusions

Although adipose tissue-induced inflammation is low-grade, it has a negative effect on distal organ function through insulin resistance, which may be responsible for complications associated with obesity. Our findings indicated that elevated white blood cell count and absolute neutrophil count in children who were overweight and obese, and especially in girls, can serve as markers of insulin resistance. In the future, further metabolomics and proteomics experiments may be able to explain the mechanism of insulin resistance in children with obesity.

## Data availability statement

The original contributions presented in the study are included in the article/**Supplementary Material**. Further inquiries can be directed to the corresponding author.

## Ethics statement

The studies involving human participants were reviewed and approved by the Ethics Committee of Henan University. Written informed consent to participate in this study was provided by the participants’ legal guardian/next of kin.

## Author contributions

LZ and H-MH contributed to the design and the data analysis of the study, and drafted the manuscript. NQ, Z-TZ, KZ, YL, H-BC, and J-NX collected and managed data. All authors approved the final article and approved the submitted version.

## Funding

This work was supported by the key scientific research project plan of Henan Province (No. 21A310006), program of interdiscipline plan based on first class disciplines (No.2019YLXKJC02), student innovation and entrepreneurship training program of Henan University (No. 20221022015, 20221022010).

## Conflict of interest

The authors declare that the research was conducted in the absence of any commercial or financial relationships that could be construed as a potential conflict of interest.

## Publisher’s note

All claims expressed in this article are solely those of the authors and do not necessarily represent those of their affiliated organizations, or those of the publisher, the editors and the reviewers. Any product that may be evaluated in this article, or claim that may be made by its manufacturer, is not guaranteed or endorsed by the publisher.
