# Supplementary material for: Insulin resistance in school-aged girls with overweight and obesity is strongly associated with elevated white blood cell count and absolute neutrophil count
Source: Front Endocrinol (Lausanne). 2022 Nov 7;13:1041761. doi: 10.3389/fendo.2022.1041761 (PMC9676363; doi:10.3389/fendo.2022.1041761)
Supplement: Supplementary file 1 [file Table_1.docx]

SUPPLEMENTARY TABLE 1 Sex differences in partial correlation analysis of BMI and clinical indicators adjusted for age in children with normal BMI

| Variables | Boy's BMI (n=96) | | Girl's BMI (n=85) | |
| --- | --- | --- | --- | --- |
|  | *r* | *P* | *r* | *P* |
| White blood cell count (10^9^/L) | 0.02 | 0.888 | 0.11 | 0.323 |
| Absolute neutrophil count (10^9^/L) | 0.09 | 0.408 | 0.14 | 0.227 |
| Fasting plasma insulin (mIU/L) | 0.29 | 0.005 | 0.28 | 0.013 |
| HOMA-IR | 0.29 | 0.005 | 0.25 | 0.027 |
| HOMA-β | 0.27 | 0.009 | 0.27 | 0.013 |
| Triglyceride (mmol/L) | 0.20 | 0.058 | 0.18 | 0.110 |
| High-density lipoprotein cholesterol (mmol/L) | -0.12 | 0.237 | -0.08 | 0.499 |
| Triglyceride/HDL ratio | 0.20 | 0.053 | 0.21 | 0.064 |
| Alanine transaminase (U/L) | 0.05 | 0.643 | 0.18 | 0.106 |
| Serum uric acid (μmol/L) | 0.05 | 0.661 | 0.26 | 0.019 |
| Systolic blood pressure (mmHg) | 0.04 | 0.720 | 0.15 | 0.194 |

BMI, body mass index; HOMA-β, homeostasis model assessment of beta-cell function; HOMA-IR, homeostasis model assessment of insulin resistance.
